# Butyrate-producing bacteria supplemented *in vitro* to Crohn’s disease patient microbiota increased butyrate production and enhanced intestinal epithelial barrier integrity

**DOI:** 10.1038/s41598-017-11734-8

**Published:** 2017-09-13

**Authors:** Annelies Geirnaert, Marta Calatayud, Charlotte Grootaert, Debby Laukens, Sarah Devriese, Guy Smagghe, Martine De Vos, Nico Boon, Tom Van de Wiele

**Affiliations:** 1 Center of Microbial Ecology and Technology (CMET), Ghent University, Coupure Links 653, 9000 Ghent, Belgium; 2 Laboratory of Food Chemistry and Human Nutrition, Ghent University, Coupure Links 653, 9000 Ghent, Belgium; 3 Department of Gastroenterology, Ghent University, 9000 Ghent, Belgium; 4 Department of Crop Protection, Ghent University, 9000 Ghent, Belgium; 5 Present Address: Laboratory of Food Biotechnology, ETH Zürich, 8092 Zürich, Switzerland

## Abstract

The management of the dysbiosed gut microbiota in inflammatory bowel diseases (IBD) is gaining more attention as a novel target to control this disease. Probiotic treatment with butyrate-producing bacteria has therapeutic potential since these bacteria are depleted in IBD patients and butyrate has beneficial effects on epithelial barrier function and overall gut health. However, studies assessing the effect of probiotic supplementation on microbe-microbe and host-microbe interactions are rare. In this study, butyrate-producing bacteria (three mono-species and one multispecies mix) were supplemented to the fecal microbial communities of ten Crohn’s disease (CD) patients in an *in vitro* system simulating the mucus- and lumen-associated microbiota. Effects of supplementation in short-chain fatty acid levels, bacterial colonization of mucus environment and intestinal epithelial barrier function were evaluated. Treatment with *F. prausnitzii* and the mix of six butyrate-producers significantly increased the butyrate production by 5–11 mol%, and colonization capacity in mucus- and lumen-associated CD microbiota. Treatments with *B*. *pullicaecorum* 25-3^T^ and the mix of six butyrate-producers improved epithelial barrier integrity *in vitro*. This study provides proof-of-concept data for the therapeutic potential of butyrate-producing bacteria in CD and supports the future preclinical development of a probiotic product containing butyrate-producing species.

## Introduction

Inflammatory bowel diseases (IBD), Crohn’s disease (CD) and ulcerative colitis (UC), are characterized by a chronic, relapsing intestinal inflammation. Although the etiology is not fully understood, research indicated that an inappropriate immune response in genetically susceptible individuals together with the complex interaction between environmental factors, intestinal microbiota and the intestinal immune system are involved in the pathogenesis of IBD^1, 2^. An influx of luminal antigens into the gut-associated lymphoid tissue, as a result of the epithelial barrier dysfunction during inflammation, continuously triggers immune cells and results in a chronic inflammation^3, 4^. Therefore, current IBD therapy is typically focused on anti-inflammatory agents and intestinal immuno-modulating therapies in order to control inflammation, restore barrier integrity and finally obtain mucosal healing^5^. Although different types of therapies are available (e.g. 5-aminosalicylates, corticosteroids, thiopurines and anti-TNF), not all are equally effective in quickly inducing prolonged remission in the entire IBD population or have side-effects, so searching for novel treatment approaches is required. The management of the gut microbiome is one of the suggested future therapies complementary to the current ones to enhance the induction and maintenance of remission in IBD^6, 7^. In fact, the growing number of human gut microbiome studies demonstrates the abnormalities in composition and functionality of gut microbiota in IBD compared to non-IBD controls. In general, microbial dysbiosis (i.e. imbalance of the microbiota) in IBD is characterized by a decrease in diversity and temporal stability of the microbiota^8–11^. At phylogenetic level, a decrease in *Firmicutes* and an increase in *Proteobacteria* taxa is the most consistent outcome from IBD microbiome studies^12^. The decreased abundance of *Firmicutes* bacteria belonging to the families *Ruminococcaceae* (also referred as clostridial cluster IV) and *Lachnospiraceae* (also referred as clostridial cluster XIVa) as opposed to healthy control samples is one of the major signatures of the microbial dysbiosis in IBD, especially in (active) CD^12–15^. Both families are important functional members of the human gut microbiota since most butyrate-producing bacteria from the human gut belong to them. The depletion of these bacterial families in IBD can be linked to the observed disturbance on a functional level, including a lower butyrate-producing capacity of the IBD microbiota^16^. In addition, a metagenomic and proteomics study in ileal CD microbiota demonstrated an underrepresentation of genes for short-chain fatty acid (SCFA) production and a decrease in metagenomic reads and proteins of important butyrate-producers *Faecalibacterium prausnitzii* and *Roseburia* sp.^17^.

Butyrate is important to maintain gastrointestinal health and has therapeutic potential in IBD, because it serves as the main energy source for colonocytes, enhances epithelial barrier integrity and inhibits inflammation^18^. Human studies have been performed in UC patients to assess the therapeutic effect of administration of pure butyrate by means of butyrate-containing tablets or enemas. However, these trials were not always successful due to delivery problems, short and discontinuous exposure of butyrate and poor compliance of the patients to the treatment^19^. An alternative approach could be the consumption of butyrate-producing bacteria to increase the *in situ* butyrate production. Therefore, it has been suggested that targeting microbial dysbiosis by supplementing butyrate-producing bacteria could restore gut homeostasis and health in IBD^20^. Such probiotic candidates are *F. prausnitzii, Roseburia spp*. or *Butyricicoccus pullicaecorum* ^20–22^. The genus *Butyricicoccus* (*Ruminococcaceae* family) is decreased in abundance in IBD fecal microbiota, its type strain, *B. pullicaecorum* 25-3^T^ is able to attenuate chemically induced colitis in a rodent IBD model^23^. We demonstrated its probiotic potential by its good intrinsic tolerance to stomach and small intestinal conditions and its potency to stimulate butyrate production by the colon microbiota *in vitro* ^24, 25^. There are a number of *in vitro* and *in vivo* studies in different cell lines and rodent colitis models demonstrating the therapeutic potential of butyrate and butyrate-producing bacteria (with focus on *F. prausnitzii*) for IBD^21, 23, 26, 27^. However, there is a lack of studies assessing the functional effect of supplementation of butyrate-producing bacteria to dysbiosed human IBD microbiota on microbial interactions (e.g. SCFA production) and host-microbe interactions (e.g. epithelial barrier integrity).

The aim of this study was to evaluate both microbe-microbe and host-microbe interactions in the microbial community of IBD patients after supplementation with butyrate-producing bacteria. CD patients were selected because there is a stronger shift in their butyrate-producing bacterial community compared to UC and healthy individuals^13^. Five CD patients with active disease and five CD patients in remission were selected to assess *in vitro* the response to treatment with butyrate-producers. We compared four types of treatment: three times a supplementation with a single butyrate-producer [*F. prausnitzii* (FP); *B. pullicaecorum* 25-3^T^ (BP25-3); *B. pullicaecorum* 1.20 (BP1.20)] and one time a supplementation with a mix of six different butyrate-producers [*B. pullicaecorum 25*-*3* ^T^, *F*. *prausnitzii*, *Roseburia hominis*, *Roseburia inulinivorans*, *Anaerostipes caccae*, and *Eubacterium hallii* (MIX)]. An *in vitro* system based on reactors resembling the lumen- and mucus-associated bacteria was used to follow up the response of CD microbiota to the treatment. *In vitro* colonization of CD fecal samples by butyrate-producers and SCFA profiles were analyzed to evaluate microbe-microbe interactions. Caco-2 monolayers were exposed to samples derived from *in vitro* CD microbiota supplemented with butyrate-producers to assess the host-microbiome interactions.

## Results

### *In vitro* incubation of fecal CD microbiota reveals differences in short-chain fatty acid production and levels of butyrate-producing bacteria

**Figure 1:**
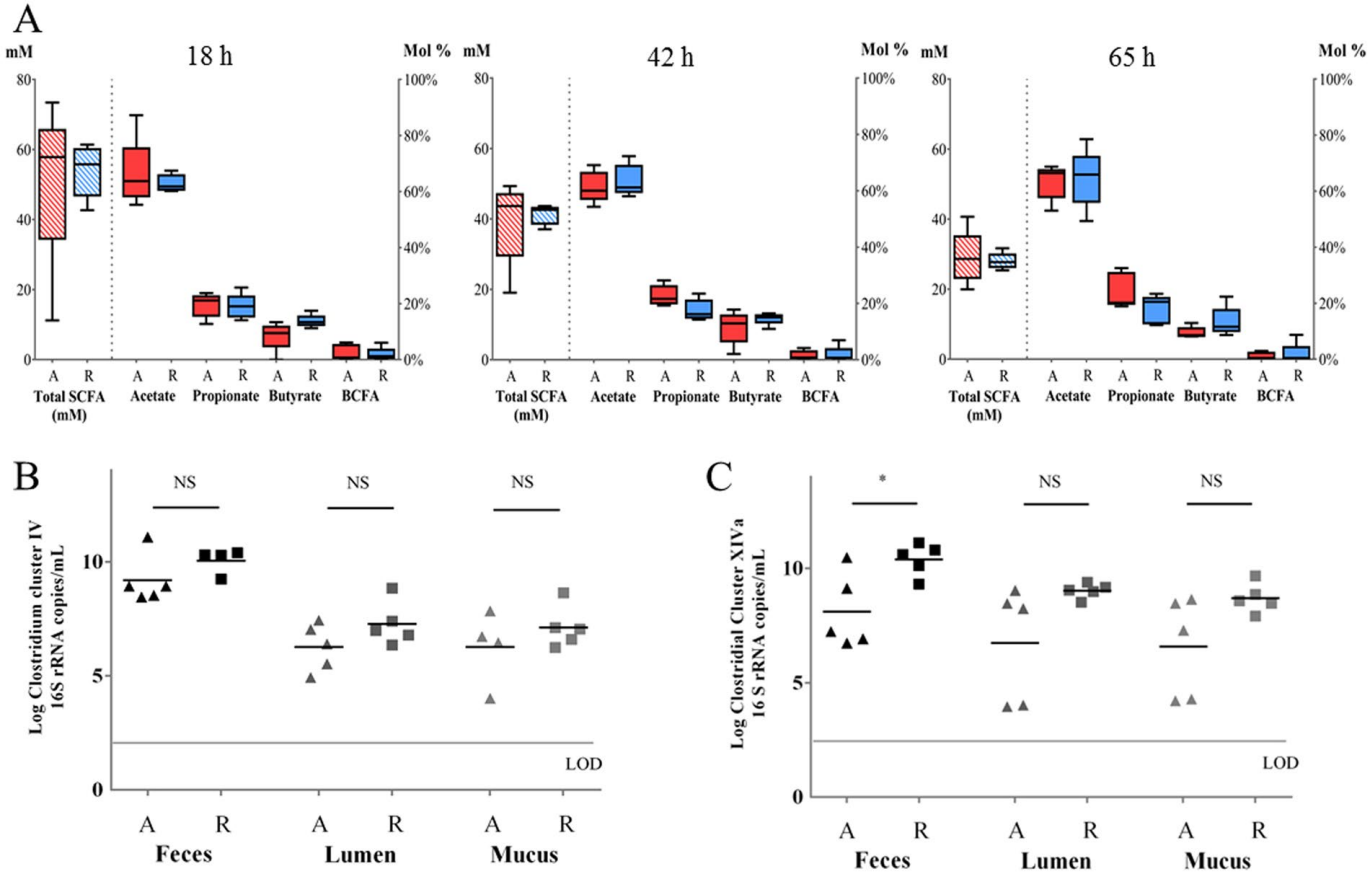
Microbial parameters of non-treated microbiota from CD patients with active disease (A; red) (n = 5) versus in remission (R; blue) (n = 5). SCFA production after 18 h, 42 h and 65 h of incubation (**A**): Boxplot of absolute SCFA concentration (downward diagonal pattern) and relative SCFA concentration (filled boxes). Black lines within boxplot represent median values and whiskers indicate minimum and maximum value. The concentration of *Ruminococcaceae* species (Clostridium cluster IV) (**B**) and *Lachnospiraceae* species (Clostridium cluster XIVa) (**C**) in fecal samples and lumen and mucus samples of non-treated microbiota after 65 h of incubation. Black lines within data plot represent mean values. Significant differences on 0.05 level between average value of active and remission samples are indicated with an asterisk.

Fecal microbiota of five CD patients with active disease and five CD patients in remission were studied during incubation for 65 h in an *in vitro* fed batch model for lumen and mucus-associated microbiota. Total SCFA production showed more inter-individual differences in active CD microbiota, which ranged from 20 to 41 mM (average 29 ± 8 mM) total SCFA at 65 h compared to remissive CD microbiota, which ranged from 25 to 32 mM (average 28 ± 2 mM) total SCFA at 65 h (Fig. 1A). Relative concentrations of acetate/propionate/butyrate showed inter-individual differences as well and were on average 63%/24%/10% (range 53–69% acetate/ 19–33% propionate/ 8–13% butyrate) for active CD and 65%/18%/13% (range 49–79% acetate/ 12–23% propionate/ 9–22% butyrate) for remissive CD microbiota at the end of incubation. There was a tendency to lower butyrate levels in active CD microbiota after 18 h, 42 h and 65 h of incubation.

Levels of *Ruminococcaceae* and *Lachnospiraceae* were monitored in fecal and *in vitro* incubated CD microbiota. Average concentrations of species belonging to *Ruminococcaceae* family were similar in active CD and remissive CD microbiota (Fig. 1B). Average concentration of *Lachnospiraceae* members was significantly lower (p = 0.02) in fecal samples of active CD (8.1 log copies/mL fecal suspension) compared to fecal samples of quiescent CD (10.7 log copies/mL fecal suspension) (Fig. 1C). At the end of the incubation, the average concentration of *Lachnospiraceae* members in non-treated samples tended to be lower in both lumen- (p = 0.08) and mucus- (p = 0.07) associated microbiota of active CD compared to remissive CD (Fig. 1C).

### Treatment with butyrate-producing bacteria results in higher butyrate production

**Figure 2:**
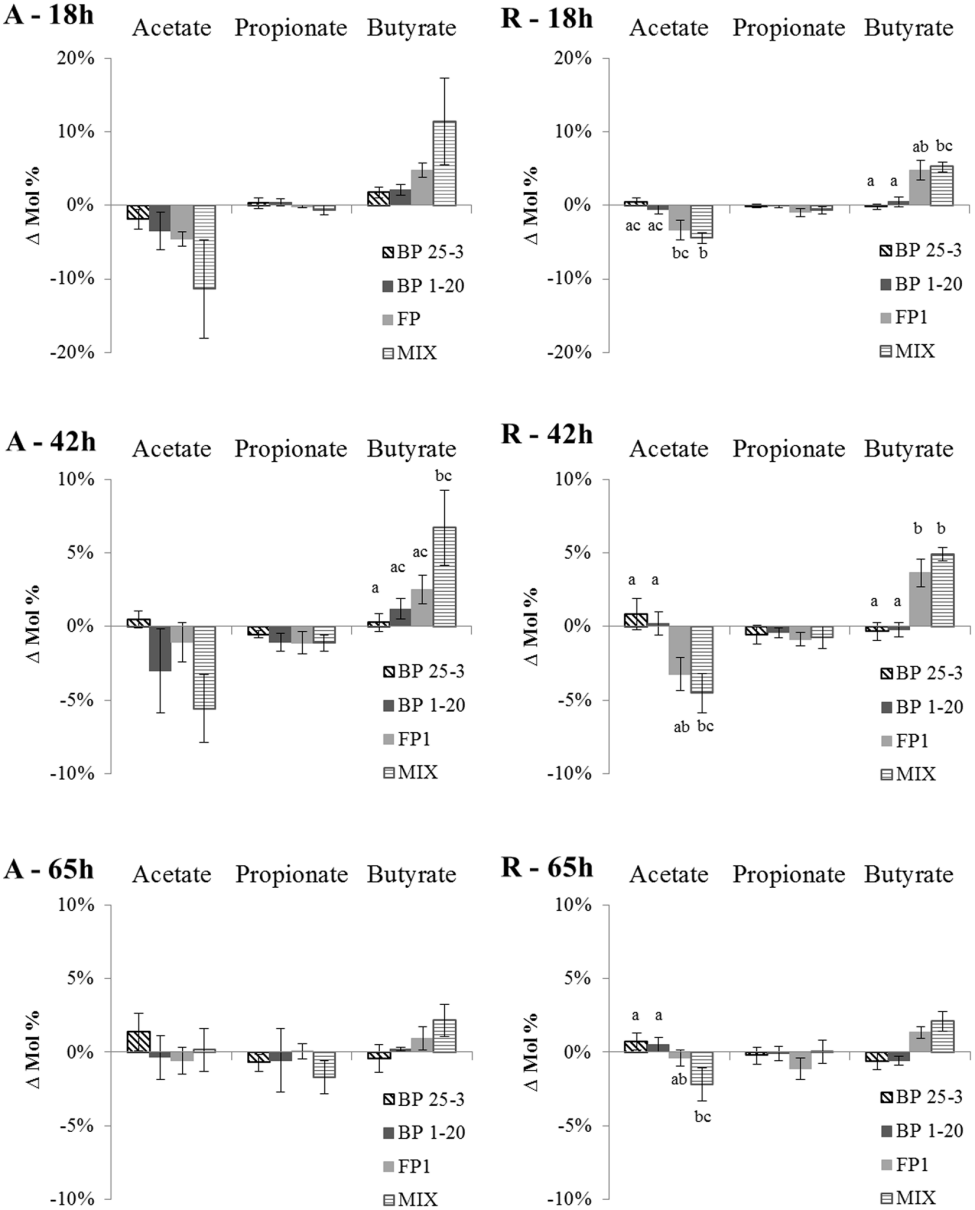
Treatment with butyrate-producers results in higher butyrate production. Δ Mol% represents the difference in SCFA levels (acetate, propionate and butyrate) between samples treated with butyrate-producers and corresponding non-treated samples. Average values and SEM are plotted for samples of active CD (A) and CD in remission (R) after 18 h, 42 h and 65 h of treatment. Treatments are indicated with letter codes: BP 25-3: *B. pullicaecorum* 25-3; BP 1-20: *B. pullicaecorum* 1.20; FP: *F. prausnitzii*; MIX: mix of *B. pullicaecorum* 25-3, *F. prausnitzii*, *R. intestinalis*, *R. hominis*, *E. hallii*, *A. caccae*. Significant differences at 0.05 level between treatments are indicated with different letter codes, the averages of bars with the same letter in their letter code are not significant different from each other.

The fecal microbiota of CD patients were supplemented with four different types of treatment containing the same number but different species of butyrate-producing bacteria. Levels of SCFAs acetate, propionate and butyrate were compared between treated and non-treated microbiota after 18 h, 42 h and 65 h of incubation (Fig. 2). Overall, there was a decrease in acetate and an increase in butyrate levels in treated microbiota. This was especially the case for treatments FP and MIX, which resulted in an average increase of butyrate production of 11% (active CD) and 5% (quiescent CD) after 18 h. Treatment with BP resulted in higher butyrate levels only in case of active CD after 18 h (BP 25-3 and BP 1.20) and after 42 h (BP 1.20). Towards the end of incubation, the difference in SCFA levels between non-treated and treated microbiota became smaller.

The different types of treatment resulted in different changes in SCFA levels. The MIX resulted in significantly higher butyrate levels and lower acetate levels compared to BP treatment in microbiota of quiescent CD (18 h and 42 h) and active CD (42 h, BP25-3, butyrate). Also, treatment with FP resulted in significantly higher butyrate levels compared to BP treatment in case of microbiota of CD in remission (42 h).

### Mix of butyrate producers results in highest colonization success

**Figure 3:**
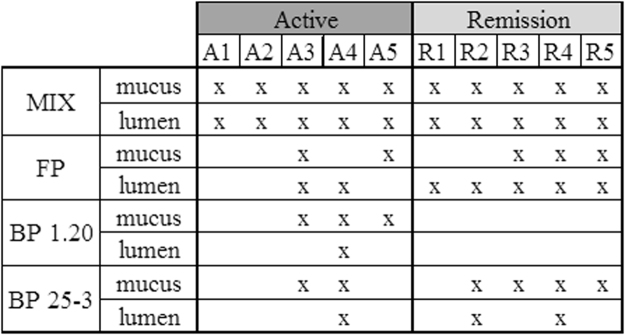
Colonization success of different treatments in mucus and lumen samples of CD microbiota (A: active; R: remission) at end of incubation. Based on group-specific DGGE profiles by comparing the relative intensity of the band classes of the different butyrate-producing species used in treatments between non-treated and treated samples. Colonization success of the supplemented butyrate-producers was defined if the relative intensity in the treated sample was higher as in the non-treated sample. In case of the MIX, there was a colonization success defined if the relative intensity of the band class of at least one species was higher in treated sample. Treatments are indicated with letter codes: BP 25-3: *B. pullicaecorum* 25-3; BP 1-20: *B. pullicaecorum* 1.20; FP: *F. prausnitzii*; MIX: mix of *B. pullicaecorum* 25-3, *F. prausnitzii*, *R. intestinalis*, *R. hominis*,* E. hallii*, *A. caccae*.

To assess the effect of supplementation of butyrate-producing bacteria on the mucus- and lumen-associated butyrate-producing bacterial communities of the five active and five remissive CD patients, we quantified and analyzed the microbial fingerprint of the families *Ruminococcaceae* and *Lachnospiraceae* by group-specific quantitative polymerase chain reaction (qPCR) and denaturing gel gradient electrophoresis (DGGE). In 40% of treated samples, the concentration of *Ruminococcaceae* and *Lachnospiraceae* members was increased compared to the control (Supplementary Table 1). Although these data suggest that in the other 60% nothing changed in both families, DGGE profiles of most treated samples showed differences with the control profiles. We introduced the parameter *colonization success* to qualitatively describe the fingerprint data (Fig. 3). In all ten incubations of CD microbiota, at least one of the butyrate-producing species of the MIX colonized the mucus- and lumen-associated butyrate-producing bacterial community. Treatment with *F. prausnitzii* resulted in a higher colonization success in the lumen- (7/10) compared to mucus- (5/10) microbiota. There was a difference in colonization success in mucus-associated butyrate-producing microbiota between both *B. pullicaecorum* strains, the 1.20 strain colonized in seven and the 25-3 strain in four out of ten cases. Their colonization success in lumen was the same, three out of ten.

**Figure 4:**
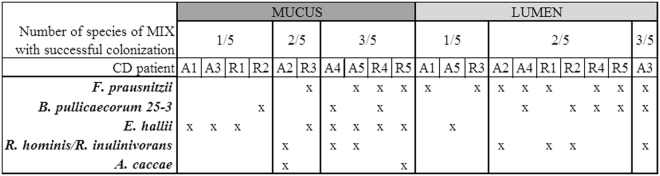
Overview of the colonization successes of the different members of the MIX in mucus- and lumen-associated butyrate-producing bacterial communities of active CD (A, n = 5) or CD in remission (R, n = 5) at the end of incubation. For each sample the number and type of butyrate-producer that colonized the microbiota is given.

Treatment with a mix of butyrate-producing bacteria resulted in all cases in a colonization success. However, it was not always the same number or same combination of species that colonized the butyrate-producing microbiota (Fig. 4). In mucus-associated butyrate-producing microbiota, *E. hallii* was the most successful colonizer (8/10) followed by *F. prausnitzii* (4/10) and *B. pullicaecorum* and *Roseburia* spp. (3/10). In lumen-associated butyrate-producing microbiota, *F. prausnitzii* was the most successful colonizer (8/10) followed by *B. pullicaecorum* and *Roseburia* spp. (5/10). Overall, *A. caccae* had the lowest colonization success as it was only present in two mucus-associated samples.

### Both *B. pullicaecorum* 25-3^T^ and a mix of butyrate producers improve epithelial barrier integrity in a Caco-2 model

The preliminary toxicity assay with 1/5, 1/10, 1/20 and 1/40 (v/v) dilutions of microbial supernatant of CD samples incubated for 42 h in fed batch system showed no cytotoxicity for 1/10 to 1/40 dilutions (data not shown). Therefore, in the following Caco-2 cell experiments 1/10 (v/v), dilutions of fed batch supernatant was used.

Caco-2 epithelial cells were grown for 7 days and treated during 11 days of differentiation with supernatant of fed batch system with microbiota of CD patients treated or not (no probiotic) with a mix of butyrate-producers (MIX) or with *B. pullicaecorum* 25-3^T^ (BP 25-3). Butyrate has a positive effect on epithelial barrier function and therefore the samples of the treatment MIX were included as these resulted in the highest increase in butyrate levels. Samples of treatment BP25-3 were included as controls for supplementation of butyrate-producers since BP25-3 did not resulted in higher butyrate levels. The epithelial barrier function was evaluated by transepithelial electrical resistance (TEER) measurement and Lucifer Yellow (LY) transport. An increase in TEER values and a decrease in LY apparent permeability (P_app_) values are indicative of a good tight junction formation and therefore a proper intestinal barrier function^28, 29^.

In general, cells exposed to CD microbial culture supernatant had significantly lower TEER values (475 ± 28 Ω cm^2^) than control cells exposed to cell culture media (632 ± 52 Ω cm^2^) (p < 0.001). However, the transport of LY showed only a trend (p = 0.057) to increase in cells exposed to CD microbial culture supernatant (2.3 ± 1.5 × 10–6 cm/s) compared to control cells (0.8 ± 0.2 × 10 6 cm/s), probably because it is a more sensitive marker of paracellular transport than TEER, thus being more affected by the interindividual variability of the CD samples.

**Figure 5:**
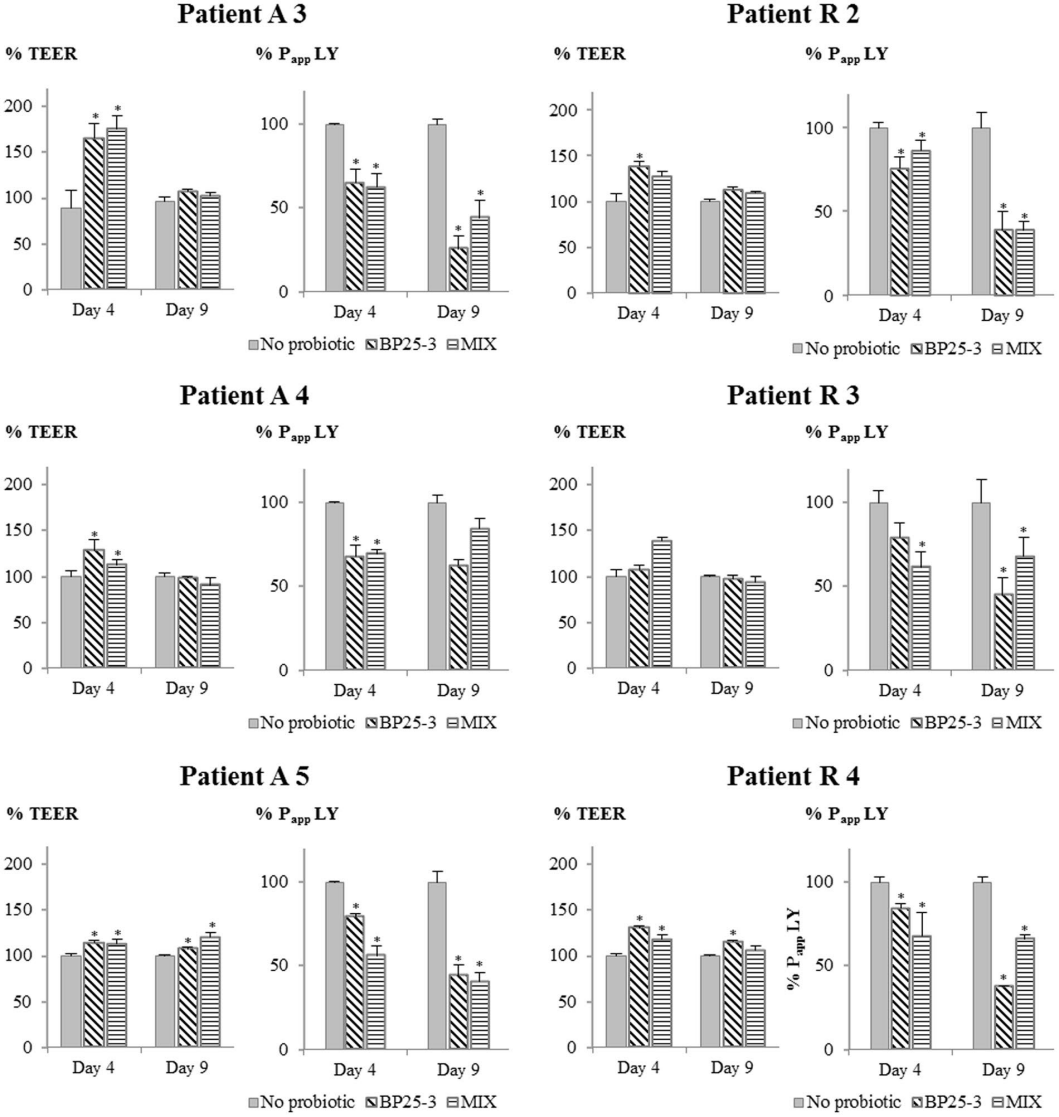
Epithelial barrier integrity at day 4 and day 9 of differentiation of Caco-2 epithelial cells treated with 1/10 (v/v) microbial supernatant of incubated CD microbiota, which were treated or not (no probiotic) with *B. pullicaecorum* 25-3^T^ (BP25-3) or a mix of six butyrate-producers (MIX). Data of BP25-3 and MIX are expressed as % of the ‘no probiotic’. Average TEER and P_app_ LY values with Stdev are shown (n ≥ 3) of the six assays (3 active (A), 3 remissive (R) patients) performed. Significant differences with corresponding ‘no probiotic’ sample are indicated with an asterisk.

Caco-2 monolayers treated with supernatant of CD microbiota treated with BP 25-3 and the MIX showed higher epithelial barrier integrity compared to non-treated microbiota (Fig. 5). All six assays taken together, the effect of increased TEER was higher after 4 days of differentiation and the effect on decreased LY P_app_ was higher after 9 days of differentiation. The TEER% of Caco-2 cells treated with supernatants from BP 25-3 varied between 107–166% (median 129%) and between 97–115% (median 108%) after 4 days and 9 days of differentiation, respectively. The TEER% of MIX varied between 113–176% (median 123%) and between 91–120% (median 104%) after 4 days and 9 days of differentiation, respectively. The P_app_ LY% of BP 25-3 treated cells varied between 65–84% (median 77%) and between 25–62% (median 41%) after 4 days and 9 days of differentiation, respectively. The P_app_ LY% of MIX treated cells varied between 56–86% (median 62%) and between 39–84% (median 55%) after 4 days and 9 days of differentiation, respectively. We observed inter-individual differences in response to treatment but no difference in treatment response between microbiota of active and remissive CD (p > 0.05). There was also no difference on the 0.05 significance level between BP25-3 and MIX for both TEER% and P_app_ LY%.

**Figure 6:**
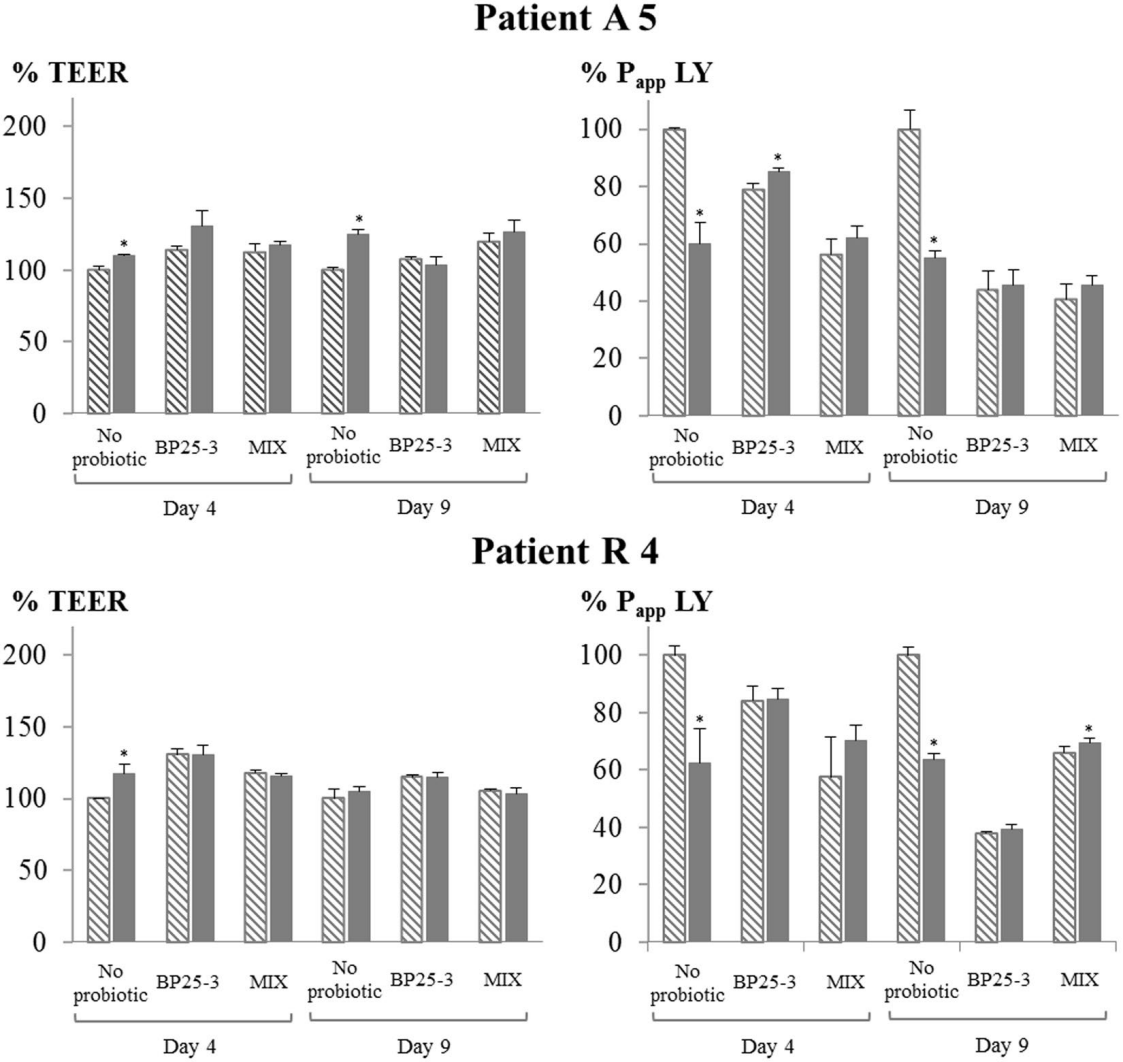
Effect of butyrate-spiking on epithelial barrier integrity. Epithelial barrier integrity at day 4 and day 9 of differentiation of Caco-2 epithelial cells treated with 1/10 (v/v) microbial supernatant of incubated CD microbiota (boxes with downward diagonal pattern), which were treated or not (no probiotic) with *B. pullicaecorum* 25-3^T^ (BP25-3) or a mix of six butyrate-producers (MIX). In addition, 1/10 (v/v) diluted supernatant samples were spiked with butyrate (final concentration of 2 mM) (dark grey boxes). Data of spiked ‘no probiotic’, BP25-3 and MIX are expressed as % of the non-spiked ‘no probiotic’. Average TEER and P_app_ LY values with stdev are shown (n ≥ 3) of the two assays (active patient 5, A5, and patient in remission 4, R4). Significant differences with corresponding ‘no probiotic’ sample are indicated with an asterisk.

To assess the epithelial barrier-improving effect of butyrate, an extra Caco-2 cell assay was performed where supernatant samples spiked with butyrate (all spiked samples contained 2 mM butyrate) were compared to non-spiked samples. Butyrate spiking of supernatant of microbiota not treated with butyrate-producing bacteria resulted in an improved epithelial barrier (Fig. 6). There was a statistically significant decrease of LY transport (both on day 4 and day 9) and an increase in TEER on day 4 of differentiation. In contrast, butyrate spiking of supernatant of microbiota treated with butyrate-producing bacteria (BP 25-3 and MIX) did not result in an improved epithelial barrier compared to non-spiked samples. In addition, butyrate-spiked control supernatant resulted in a similar or even lower epithelial barrier integrity as non-butyrate-spiked BP25-3 or MIX supernatants.

## Discussion

The aim of this study was to demonstrate the positive functional effects of supplementing butyrate-producing bacteria to a dysbiosed microbial community by assessing the microbe-microbe and host-microbe interactions *in vitro*. With these results we wanted to support the rationale to use butyrate-producing bacteria as a probiotics to target microbial dysbiosis in IBD. Patients with CD (active and remission) were selected as there is a major shift in their butyrate-producing gut microbial community, especially in active CD^13^.

Supplementation of butyrate-producing bacteria to fecal microbiota of five active and five remissive CD patients resulted in increased butyrate and decreased acetate levels during the 65 h incubation upon supplementation. The decrease in acetate can be explained by metabolic cross-feeding between acetate-producing and butyrate-producing bacteria. During butyrate synthesis, butyryl-CoA is converted to butyrate by either butyryl-CoA:acetate CoA-transferase using acetate as co-substrate or by butyrate kinase after butyryl-CoA phosphorylation^30^. The former is the dominating route for butyrate production by human colon bacteria^30, 31^ and it was shown that the supply of exogenous acetate is important to maintain butyrate production^32^. In this research, the decrease in acetate levels and the subsequent increase in butyrate is supportive of the butyryl-CoA:acetate CoA-transferase route. Although the number of supplemented butyrate-producers in the four different treatments was the same, only FP and MIX resulted in an increased butyrate production, which was still detectable at the end of incubation. This indicated that *F. prausnitzii* A2–165 and one or more members of the mix of butyrate-producers were able to compete more successful than *B. pullicaecorum* with the other microbiota for the available substrates resulting in butyrate production. For example, one of the carbon sources in the simulation medium was apple pectin and it was shown that *F. prausnitzii* A2–165 is able to utilize it as sole energy source and produce butyrate from it^33^. The capacity of fermenting apple pectin is not a common feature among butyrate-producing *Firmicutes* bacteria, as so far, there are only two other pectin-utilizing *Firmicutes* species identified (*Eubacterium eligens* and *Lachnospira pectinoschiza*)^34^. A fermentation study by Moens and co-workers with fructose, oligofructose or long-chain inulin as sole carbon source, showed that *B. pullicaecorum* 25-3^T^ could only degrade and produce butyrate from fructose indicating it relies on other species to degrade complex substrates to simple sugars. In contrast, *F. prausnitzii* A2–165 was able to also degrade complex substrates, oligofructose and inulin, resulting in butyrate production^35^. As substrate degradation is very species specific, the use of a mix of different butyrate-producers increases the chance that at least one of the members is able to compete with the resident microbiota for available substrates, resulting in colonization, growth and butyrate production.

In addition to the species-specific functionalities, the impact of intra-species diversity needs to be considered. For example, Lopez-Siles *et al*. described two different phylogroups of *F. prausnitzii* ^33^. While phylogroup I is decreased in CD, UC, and colorectal cancer, depletion of phylogroup II was specifically related to CD^36^. In this study, the CD microbiota was amended with *F. prausnitzii* A2–165 type strain (phylogroup II), supporting our hypothesis of supplementing functional bacteria that are depleted in CD for enhancing butyrate production. Therefore, only the specific fingerprint of the supplemented *F. prausnitzii* (phylogroup II) was considered in the *Ruminococcaceae* bacterial fingerprints in our study during the evaluation of the colonization success.

The supernatant of CD microbiota supplemented with *B. pullicaecorum* 25-3^T^ or a mix of six butyrate-producers improved the epithelial barrier integrity in a Caco-2 Transwell model demonstrated by increased TEER and decreased paracellular flux of LY. Of the *in vitro* models, Caco-2 cells are one of the most widely used to study epithelial barrier function as these cells differentiate spontaneously in culture, leading to development of tight junctions (TJ) and apical-basolateral cell polarity. Both TEER and LY-flux are measures for the monolayer integrity and formation and function of TJ structures between the Caco-2 cells. However, LY-flux was more sensitive as the differences in P_app_ LY with non-treated samples were much higher compared to TEER differences. Certain microbial compounds or metabolites in the BP25-3- and MIX-treated microbial supernatants could have an influence on the epithelial barrier integrity. As a matter of fact, the role of microbial butyrate in improving epithelial barrier function is well known and demonstrated in a number of *in vitro* and *in vivo* studies^37^. At concentrations at the same level of our butyrate-spiking assay (2 mM), butyrate promoted expression of tight junction proteins and facilitated their assembly by acceleration of the relocation of zonulin-1 and occludin, which resulted in increased barrier integrity^38, 39^. This explains the observed increase in epithelial barrier integrity after spiking of the supernatants of microbiota not treated with butyrate producers. However, butyrate-spiking of BP25-3 and MIX supernatants did not result in an additional improvement of epithelial barrier integrity. Moreover, butyrate and SCFA concentrations in supernatants of microbiota treated with *B. pullicaecorum* 25-3^T^ were not higher as in supernatants of non-treated microbiota. So butyrate is not the only microbial factor that can explain the improved epithelial barrier.

The effect of culture supernatant of *B. pullicaecorum* 25-3^T^ in regulating the epithelial barrier function was already demonstrated in two previous studies with single cultures of *B. pullicaecorum*. It was also able to preserve epithelial barrier function and down-regulate IL-8 production in TNFα and IFNγ stimulated Caco-2 cells^23^. In a follow-up study, it was demonstrated that the concentration of *Butyricicoccus* spp. negatively correlates with claudin-1 expression in the mucosa of active UC patients and that *B. pullicaecorum* culture supernatant normalized claudin-1 expression in inflamed UC biopsies^40^. Although most TJ proteins are decreased in IBD, TJ protein claudin-1 is increased in inflamed areas in IBD^41^. Therefore, it is hypothesized that *B. pullicaecorum* 25-3^T^ can regulate the TJ structures leading to a proper epithelial barrier integrity. It has been described that other microbial metabolites, as indole 3-propionic acid^42^ or 10-Hydroxy-cis-12-octadecenoic acid^43^ are involved in regulation of epithelial barrier function and tight junction expression. So, to elucidate the mechanism of host and *B. pullicaecorum* 25-3^T^ interactions further studies are necessary.

The Caco-2 cell line differentiated in semipermeable membranes is the standard model for studying epithelial barrier function since these cells develop functional TJ complexes to a higher degree than monolayers of other intestinal epithelial cell lines (e.g. HT29-MTX)^44^. However, in most assays, treatment is applied to differentiated cells for a short period (24 h) after disruption of barrier integrity. In our set-up, we evaluated the effect of our treatment on pre-differentiated cells (7 days after seeding) throughout the development of epithelial barrier integrity (11 days). This is a unique approach which has, as far as we know, only been presented by Van Rymenant *et al*.^45^. They have demonstrated significant differences in TEER development and polyphenol metabolism and transport when Caco-2 cells were pretreated with a 1/20 dilution of filter sterilized microbial suspension for 14 days, compared to a single exposure of non-pretreated cells. The exposure of Caco-2 cells to bacterial metabolites during the time of differentiation resembles, in a more realistic way, the complex environment at the intestinal level.

Similarly, in this study microbial supernatant and not living bacteria were applied with the rationale that *in vivo*, direct contact between intestinal epithelial cells and microbiota is minimal since the epithelium is covered by a mucus layer. Even in CD, not a thinner, as in UC patients, but a thicker mucus layer with altered structure is present^46^. Also, our set-up differed from that of others by the indirect treatment-effect of probiotic candidates. Previously published *in vitro* studies evaluated probiotic candidates by applying (culture supernatant of) the probiotic candidate to epithelial cells^47^. This research applied a different approach, in which probiotic candidates were supplemented to microbiota of CD patients, allowing microbe-microbe interactions during incubation. Furthermore, the complex microbial supernatant was used to treat Caco-2 cells for a long-term (11 days), providing a more realistic environment to simulate probiotic treatment.

Supplementation of butyrate-producing bacteria to CD microbiota resulted in changes in the microbial fingerprint of mucus- and lumen-associated butyrate-producing microbiota at the end of the incubation. Overall, a mix of six butyrate-producers had the highest colonization success rate since at least one species colonized all CD butyrate-producing microbiota and in some cases, three species colonized. The type and combination of colonizing species varied among the different CD microbiota, which indicates that the mix can adapt to the endogenous microbial community. Several other studies provided evidence that multispecies probiotic formulations are more effective than monostrain probiotics, e.g. in preventing antibiotic-associated dysbiosis in children^48, 49^. However, the underlying mechanism of the better performance of the multispecies mix of butyrate-producers is complex and further research is needed to reveal possible synergistic interactions between members of the mix.

So far, microbiota modulation in IBD by probiotics showed low success in controlling the disease and probiotic effectiveness to induce and maintain remission was only shown for a UC scenario^50^. This can be due to the experimental set-up of the different trials (dose, formulation, design of the trial, measured end points, small number and heterogeneity of patients) but can also be caused by selected probiotic species^51^. For example, none of the trials with probiotics in IBD included butyrate-producing bacteria. Another promising strategy to modulate the microbiota in IBD is transfer of fecal microbiota (FMT) of a healthy donor^52^. A meta-analysis of FMT studies in IBD showed, in contrast to probiotics, a higher clinical response in patients with CD (61%) compared to UC (22%)^53^. Efficacy of FMT is dependent on the composition and functionality of the donor microbiota. Donors with a higher proportion of *Lachnospiraceae* bacteria resulted in a higher success rate of FMT in IBD^54^. In addition, recipients that responded to FMT had an increase in *Lachnospiraceae* or *Ruminococcaceae* species post-FMT^55–58^. These findings support the hypothesis and results of our study that butyrate-producing *Lachnospiraceae* and *Ruminococcaceae* bacteria are essential members of future microbial-based products for controlling CD.

While the supplementation of butyrate-producing clostridia displayed promising data regarding strengthening of the epithelial barrier functioning, our experiments were only conducted with the simulated gut microbiota from 10 Crohn’s disease patients. While our data were very consistent, the biological reproducibility needs to be further tested with simulated gut microbiota from more patients and *in vivo* experiments in CD animal models. This will further support the robustness of probiotic strategies like these towards future applications aiming to enhance epithelial barrier function or prolonging or even inducing remission in chronic gut inflammation.

To conclude, this study demonstrated that *in vitro* supplementation of microbiota of CD patients with butyrate-producing bacteria results in a higher butyrate production. A multispecies mix of butyrate-producers had the highest success rate in terms of increased butyrate production and colonization capacity in mucus- and lumen-associated CD microbiota. In addition, we have demonstrated the capacity of butyrate-producing bacteria and especially of *B. pullicaecorum* 25-3^T^ to improve epithelial barrier integrity, which was not only a consequence of their butyrate production. Future studies will have to elucidate the mechanisms of action and identify the metabolite/s or bio-active compound/s that improve intestinal epithelial barrier integrity. The capacity of improving epithelial barrier integrity may have important therapeutic consequences for CD as a disrupted intestinal barrier function is associated with development of the disease.

## Methods

### Bacterial strain, growth conditions and preparation treatment

Butyrate-producing bacteria *Butyricicoccus pullicaecorum* 25-3^T^ (LMG 24109 ^T^), *Butyricicoccus pullicaecorum* 1.20 (strain isolated from human feces, kindly provided by the Department of Pathology, Bacteriology and Avian Diseases of Ghent University), *Faecalibacterium prausnitzii* (DSM 17677), *Roseburia hominis* (DSM 16839), *Roseburia inulinivorans* (DSM 16841), *Anaerostipes caccae* (DSM 14662) and *Eubacterium hallii* (DSM 3353) were grown in anaerobic M2GSC medium at pH 6 prepared as described by Miyazaki *et al*.^59^ but with 15% (v/v) of clarified rumen fluid instead of 30% (v/v). M2GSC agar (1.5% m/v) plates were incubated at 37 °C in an anaerobic (10% CO_2_, 90% N_2_) workstation (GP-Campus, Jacomex, TCPS NV, Rotselaar, Belgium) for 20–40 h. Before use in each experiment, a colony of each butyrate-producing species was transferred into 10 mL of anaerobic M2GSC broth and incubated overnight at 37 °C. Subsequently, the culture was subcultured (1% v/v) once in anaerobic M2GSC broth and incubated for 20 h at 37 °C. The cultures were concentrated 20 times by centrifugation (10 min, 1500 g). The supernatant was removed and the pellet was suspended in anaerobic nutritional medium (see below). Concentration of total and intact bacteria in suspension was determined by means of fluorescent staining (SYBR Green/ propidium iodide) (Invitrogen) and flow cytometry according to Van Nevel *et al*.^60^. Staining procedure was adjusted to 4 µM propidium iodide and 13 min incubation at 37 °C.

### Fecal bacteria from CD patients

Fecal samples were obtained from 10 CD patients to use for incubation in a fed batch system. The study was approved by the Ethics Committee of the University Hospital Ghent (permit numbers EC UZG 2006/377 & EC UZG 2012/415), and all volunteers received and signed an informed consent form. The methods used in this study were carried out in accordance with the relevant guidelines and regulations, including any relevant details.

None of the patients received antibiotics or probiotics for at least 3 months and a colonic lavage preparation for colonoscopy at least 1 month before fecal sample donation. Five patients were in clinical remission and five had active disease, determined by presence of endoscopic signs of disease activity after fecal sample donation. Patient metadata including age, gender, disease location, inflammatory markers and medication is listed in Supplementary Table 2.

Fresh fecal samples were collected in airtight containers together with one AnaeroGen sachet (Oxoid Ltd, Hampshire, UK) to obtain and maintain anoxic conditions until start of incubation. Time between fecal sample donation and start of incubation was maximum 4 h. A 20% (m/v) fecal suspension was prepared by homogenizing the fecal sample with 0.1 M anaerobic phosphate buffer (per L: 8.8 g K_2_HPO_4_, 6.8 g KH_2_PO_4_ and 1 g C_2_H_3_O_2_SNa, pH 6.8) in a stomacher for 2 min. After removing particulate material by centrifugation (2 min at 500 g), the suspension was used as inoculum for incubation.

### Fed batch incubations - Simulation of lumen- and mucus-associated microbiota

Fecal microbiota were incubated for 65 h in a fed batch system for simulation of proximal colon conditions and consisted of 250 mL airtight bottles flushed with N_2_ and filled with 100 mL nutritional medium and 16 mucin agar-covered microcosms in a polyethylene netting. Nutritional medium contained per L: yeast extract (3 g) (Oxoid); special peptone (1 g) (Sigma); mucin (2 g) (Sigma); arabinogalactan (0.25 g) (Sigma); apple pectin (0.5 g) (Sigma); xylan (0.25 g); starch (1 g) (Sigma) and the medium was buffered by addition of KH_2_PO_4_ (9.53 g) and Na_2_HPO_4_.2H_2_O (5.34 g) (Sorensen’s phosphate buffer, pH 6.47) (Carl Roth GmbH + Co KG, Germany). The mucin-agar beads served as a glycoprotein contact surface which resulted in a better simulation of the colon microbiota^61, 62^. Mucin agar consisted of 5% (w/v) commercial pig gastric mucin (Sigma) and 1% (w/v) agar (Sigma). Bottles were incubated at 37 °C in the dark and under slow shaking. Twice a day, after 18 h, 26 h, 42 h and 50 h of incubation, 40% (v/v) of the bacterial suspension was replaced by sterile, anaerobic simulation suspension. Simulation suspension consisted of 70% (v/v) sterile nutritional medium and 30% (v/v) pancreatic simulation fluid [per L: 6 g dehydrated bile extract (Oxgall, Difco) and 0.9 g pancreatin (Sigma)]. Every day, pH of the microbial suspension was measured to control proper functioning of the buffer, the pH ranged from pH 5.88–6.62 with median at pH 6.23.

At time of start-up, fecal microbiota were supplemented to the fed-batch system at a final concentration of 1.5% (w/v) and supplemented with butyrate-producing bacteria. There were four different treatments: *B. pullicaecorum* 25-3^T^ (BP 25-3); *B. pullicaecorum* 1.20 (BP 1.20), *F. prausnitzii* (FP) and a mix of *B. pullicaecorum* 25-3^T^, *F. prausnitzii*, *R. hominis*, *R. inulinivorans*, *A. caccae* and *E. hallii* (MIX) and in parallel a control with only fecal microbiota. In each incubation and type of treatment the same concentration of butyrate-producing bacteria was applied, based on flow cytometry results, of 6.74*10^8^ viable (intact) bacteria/mL fed batch medium or 6.98*10^8^ total bacteria/mL fed batch medium. The mix of butyrate-producing bacteria was prepared by adding the separate stock suspensions of each species. Each treatment and control fed batch incubation for the 10 CD patients was done in duplicate (technical replicate).

Lumen samples were taken every day, mucin agar samples were taken at the end of incubation (65 h). Mucin agar-covered microcosms were washed with sterile PBS to remove luminal bacteria. Mucin agar was then removed from the microcosms, homogenized and stored immediately at −20 °C in aliquots of 0.25 g until further analysis. A fraction of the lumen samples was prepared to use for Caco-2 cell experiments. Therefore, samples were centrifuged for 10 min at 1500 g, supernatant was collected and filter-sterilized over a 0.22 µM PVDF syringe filter (Merck Millipore, Darmstadt, Germany) and immediately stored at −80 °C in aliquots of 1 ml.

### Short-Chain Fatty Acids analysis

The SCFA in the luminal samples were extracted with diethyl ether and analyzed using a gas chromatograph as described by De Weirdt *et al*.^63^. The concentration in mM of acetate, propionate, butyrate, isobutyrate, valerate, isovalerate, caproate and isocaproate was determined in each sample. The concentration of acetate, propionate, butyrate and total branched SCFA (BCFA) (sum of isobutyrate, isovalerate and isocaproate) was expressed as mol% which is the ratio of their concentration (mM) and the total SCFA concentration (mM) multiplied by 100 in the sample.

### Microbial community analysis

#### DNA extraction

Liquid samples (1 mL fecal suspension and 1 mL lumen) for total DNA extraction were centrifuged for 10 min at maximum speed, supernatant was removed and pellet was stored immediately at −20 °C until further analysis.

Total DNA was extracted from pellet of liquid samples and 250 mg mucin agar using a phenol-chloroform extraction protocol as previously described^25^. After finishing the extraction protocol, DNA samples were immediately stored at −20 °C until further analysis. Quality of DNA samples was analyzed by 1% (w/v) agarose (Life Technologies, Madrid, Spain) gel electrophoresis.

#### PCR-DGGE

To analyze the microbial community in fecal, lumen and mucin-agar samples, PCR was performed with group specific primers with GC-clamp to amplify a 16 S rRNA gene fragment of *Ruminococcaceae* and *Lachnospiraceae* families. Details of primers are listed up in Supplementary Table 3. PCR amplicons were separated by DGGE using an Ingeny phorU2 × 2 DGGE-system (Ingeny, Goes, the Netherlands) for total and *Lachnospiraceae* bacteria and a D-code DGGE system (Bio-Rad, Nazareth, Belgium) for *Ruminococcaceae*. Details on each DGGE protocol are included in Supplementary Table 4. After electrophoresis, gels were stained for 20 min in dark in a 33x SYBR Green (Life Technologies, Invitrogen) 1x Tris-Acetate-EDTA buffer (Applichem). Stained gels were immediately photographed on a UV-transillumination table with camera (OptiGo 600, Isogen) and software ProXima AQ-4 (Isogen Life Sciences, the Netherlands).

Analysis of the fingerprint data was done with BioNumerics software version 5.10 (Applied Maths, Sint-Martens-Latem, Belgium). Different lanes of each gel were defined, background was subtracted, gel was normalized using a home-made marker lane and bands were detected (intensity higher than 1%). To monitor the different supplemented butyrate-producing bacterial species in the fingerprint profiles, band-class analysis was performed. With BioNumerics bandmatching-tool, band-classes were identified over all profiles for group-specific DGGE profiles of *Ruminococcaceae* and *Lachnospiraceae* as described earlier^64^. Each band in the fingerprint profile was designated to a band-class based on their relative position within the fingerprint profile. On every gel there were lanes with PCR amplicons of each supplemented butyrate-producing species included. The band class of the different butyrate-producing species was used to identify band classes in the profile of lumen and mucin-agar samples. The relative intensity per band class was exported and used to compare relative intensities of the different butyrate-producers between non-treated and treated samples. A ‘colonization success’ of the supplemented butyrate-producers was defined when the relative intensity in the treated sample was higher as in the non-treated sample.

#### qPCR

Group specific 16 S rRNA gene qPCR analysis was performed to quantify *Ruminococcaceae* and *Lachnospiraceae* species in DNA extracts of fecal (1000-fold diluted) and lumen and mucin-ager samples (10-fold diluted) (Supplementary Table 1). All qPCR assays were performed on a StepOnePlus^TM^ Real-Time PCR system (Applied Biosystems, Carlsbad, CA). The amplification reactions were carried out in triplicate in a volume of 12.5 µL which contained 10 µL of Power SYBR Green mastermix (Applied Biosystems, Foster City, CA) and 2.5 µL of DNA template. The cycling program for each protocol was as follows: 10 min at 95 °C followed by 40 cycles of 15 s at 95 °C, 1 min at 50 °C and 30 s at 72 °C. Primer specificity and verification of presence of desired amplicon was determined by melting curve analysis and gel electrophoresis. For each qPCR assay, standard curves were created by a 10-fold dilution series of plasmid DNA containing the 16 S rRNA gene fragment of *B. pullicaecorum* 25-3^T^ (clostridial cluster IV assay) or *Roseburia* sp. (clostridial cluster XIVa assay). PCR efficiency (%) was calculated from the slope of the standard curve of each qPCR assay (10^(−1/slope)^ – 1). Assays with an efficiency of 80–110% (slope of 3.2–3.9) were included in data analysis.

### Assessment of epithelial barrier function in response to BP 25-3 and MIX treatment: Caco-2 cell experiments

#### Cell cultures

Caco-2 is the most widely used immortalized cell line for developing human gastrointestinal tract in *in vitro* models. This cell line spontaneously differentiate into polarized cells with distinct mucosal (apical) and serosal (basolateral) cell membrane domains, brush border enzymes and polarized expression of transporters^65–67^.

The Caco-2 cells were obtained from the American Type Culture Collection (ATCC^®^ HTB-37™ATCC; LGC Standards, France). Cell maintenance was carried out in 25 cm^2^ flasks to which 4 mL of Dulbecco’s Modified Eagle Medium with high glucose (4.5 g/L) and GlutaMAX™ (Gibco, Langley, OK, USA) (DMEM). The DMEM was supplemented with: 10% (v/v) heat-inactivated fetal bovine serum (FBS) (FBS, Greiner Bio-One, Wemmel, Belgium), 1% non-essential amino acids, and 2% penicillin/streptomycin (Life Technologies, Merelbeke, Belgium) to obtain complete cell growth medium. Medium was refreshed every two days and cells were subcultured when they reached 80% confluence. Briefly, Caco-2 cells were detached with a pre-wash with phosphate buffer saline without calcium and magnesium (PBS, Gibco), trypsinized for 5–8 min with trypsin solution (2.5 g/L) and ethylenediaminetetraacetic acid (EDTA, 0.2 g/L) (Gibco) and neutralized by the addition of complete medium, followed by reseeding at a density of 5 × 10^4^ cells/cm². The cell morphology was analyzed by phase-contrast microscopy (Motic AE31, VWR, Leuven, Belgium). The cells were incubated at 37 °C in an atmosphere with 95% relative humidity and a CO_2_ flow of 10%. All the cultures were used between passages 6 and 10.

#### Toxicity assay of microbial culture supernatant from incubated CD microbiota on Caco-2 epithelial cells

To determine the potential cytotoxic effect of the supernatant samples from fed batch assays on Caco-2 cells and to establish sub-toxic conditions to perform the tests on Transwell^®^ inserts, different dilutions of the supernatant samples were evaluated. Supernatants of fed batch incubation of microbiota from two patients (one active and one in remission and control samples) were diluted 1/5, 1/10, 1/20 and 1/40 (v/v) in DMEM medium. In addition, different butyrate concentrations were tested (2 mM, 4 mM, 8 mM and 12 mM). Caco-2 cells were seeded in 96 well plates in a density of 7.5 × 10^4^ cells/cm^2^. After 2 weeks of differentiation the cells were stimulated with 0.2 mL of diluted supernatant samples or butyrate suspension.

Cellular activity, viability and protein content were measured after 24, 48 and 72 h of exposure to treatment. The effect on cellular activity was evaluated by using the MTT (3-[4,5-dimethylthiazol-2-yl]−2,3-diphenyl tetrazolium bromide) assay (Sigma)^68^, a yellow solution which is converted to blue formazan crystals by mitochondrial activity. MTT solution was added at a final concentration of 0.5 mg/mL into the cell culture medium and incubated at 37 °C for 2 h. Afterwards, the medium was removed, and the formazan crystals were dissolved in 200 µL dimethyl sulfoxide (DMSO) and measured using a spectrophotometer (Benchmark Plus Microplate Reader, Bio-Rad, Hercules, CA) at a wavelength of 530 nm. Unspiked control cells exposed to cell culture media were used throughout each assay. The SRB assay (sulforhodamine B) was used for the measurement of cellular protein content, as described earlier^69^. Briefly, cells were fixed by adding trichloroacetic acid (final concentration 25%) for 1 h, washed with tap water, treated with SRB stain for 30 min, washed again with 1% glacial acetic acid to remove the excess of stain, suspended in Tris-buffer and measured at 490 nm. Membrane integrity was monitored by the trypan blue exclusion assay^70^. Cells were harvested using trypsin–EDTA solution (2.5 g/L trypsin, 0.2 g/L EDTA), collected and mixed with an equal volume of PBS containing 0.4% (w/v) trypan blue dye and then microscopically counted with a Bürker counting chamber. Six replicates were made for each treatment for MTT, SRB and membrane permeability test. Cytotoxicity was expressed as percentage of value for the untreated cells exposed to the cell culture media.

#### Effect of microbial culture supernatant from CD microbiota treated with butyrate-producers on epithelial barrier function

Cell differentiation and the posterior tests were carried out in double chamber wells (Corning^®^ HTS Transwell^®^−24 well, pore size 0.4 µm; Costar, NY) equipped with separate apical and basolateral compartments and a porous support on which the Caco-2 cells can grow into a monolayer. The cells were seeded at a density of 7.5 × 10^4^ cells/cm² and maintained with complete DMEM until confluency (1 week). The volumes used for the apical and basolateral compartments were 0.2 and 1 mL respectively. One week after seeding, the complete medium was removed and cells were washed twice with DMEM without supplementation. The supernatants of fed batch systems from 6 CD patients (3 in remission, 3 with active disease) treated or not with butyrate-producing bacteria (BP 25-3 or MIX) were diluted 1/10 in DMEM and adjusted to pH 7.2 with NaOH (0.5 M). An overview with characteristics (pH and SCFA concentrations) of the undiluted supernatant samples used in this assay is given in Supplementary Table 5. Next, 0.2 mL of filtered supernatant was added to the apical chambers. Different treatments from each patient were evaluated independently, at least in triplicate. One mL of DMEM supplemented with 20% of FBS was added to the basolateral compartment. Every two days until day 19 after seeding, apical and basolateral media were replaced and the cell monolayer integrity was evaluated.

#### Measurement of epithelial barrier function: transepithelial electrical resistance (TEER) and apparent permeability (Papp) of paracellular marker

The monolayer integrity was assessed by measuring the TEER and P_app_ of the paracellular transport marker lucifer yellow (LY, Sigma-Aldrich, Belgium). An automated TEER Measuring System with a REMS auto-sampler (World Precision Instruments, Berlin, Germany) was used for the TEER measurements. During the growth period (1 week after seeding), the cell monolayer status was evaluated every two days. The cell monolayer was considered to be confluent when stable values of ≥350 Ω cm^2^ were obtained and only monolayers in this condition were used. P_app_ of LY, which is mainly transported via the paracellular route, was used to assess the integrity of the epithelial cell monolayer. P_app_ of LY was measured by adding the marker (100 µM) to the apical compartment of the wells. After 15, 30, 60 and 120 min, 100 µL of medium was removed from the basolateral compartment and replaced with an equal volume of fresh medium (DMEM with 20% FBS). LY fluorescence was measured at an excitation/emission wavelength of 485/520 nm in 96 black plates (Greiner), using a microplate fluorescence reader (Spectramax Gemini XS Microplate Reader, Molecular devices, Orleans, CA). A calibration curve (0, 5, 10, 25, 50 and 100 µM) for LY quantification was run in duplicate in each reading. The P_app_ coefficients were calculated as previously described^71^. Only monolayers with P_app_ values < 0.1 × 10^−6^ cm/s at the end of the assay were included in data-analysis. TEER and P_app_ results of epithelial cells treated with BP 25-3 and MIX samples were normalized to the results of cells treated with corresponding control sample (fecal microbiota, but no probiotic-butyrate-producer added) and expressed as %.

### Statistical analysis

All statistical analysis were performed in SPSS Statistics 21 software (SPSS, Chicago, IL). Significance level was set at 0.05. Normality of the data set was tested with the Kolmogorov-Smirnov test. In case of normality, mean values of two different groups were compared with an independent samples t-test. Significant differences between different treatment on SCFA levels were tested with one way ANOVA in case of normality. Homogeneity of variances was tested with the Modified Levene test. Depending on the outcome of the Levene test, Bonferroni or Dunett T3 were used as post hoc tests to determine p-values. In case of non-normal distributions, differences were tested with non-parametric Mann-Whitney U test.

## Electronic supplementary material

Supplementary information
