# Supplementary material for: Butyrate-producing bacteria supplemented in vitro to Crohn’s disease patient microbiota increased butyrate production and enhanced intestinal epithelial barrier integrity
Source: Sci Rep. 2017 Sep 13;7:11450. doi: 10.1038/s41598-017-11734-8 (PMC5597586; doi:10.1038/s41598-017-11734-8)
Supplement: Supplementary file 1 — Supplementary information [file 41598_2017_11734_MOESM1_ESM.pdf]

SUPPLEMENTARY INFORMATION

Butyrate-producing bacteria supplemented *in vitro* to Crohn's disease patient microbiota increased butyrate production and enhanced intestinal epithelial barrier integrity

Annelies Geirnaert [a, b†], Marta Calatayud [a†], Charlotte Grootaert [c], Debby Laukens [d], Sarah Devriese [d], Guy Smagghe [e], Martine De Vos [d], Nico Boon [a] and Tom Van de Wiele [a*]

[a] Center of Microbial Ecology and Technology (CMET), Ghent University, Coupure Links 653, 9000 Ghent, Belgium;

[b] Current address: Laboratory of Food Biotechnology, ETH Zürich, 8092 Zürich, Switzerland;

[c] Laboratory of Food Chemistry and Human Nutrition, Ghent University, Coupure Links 653, 9000 Ghent, Belgium;

[d] Department of Gastroenterology, Ghent University, 9000 Ghent, Belgium

[e] Department of Crop Protection, Ghent University, 9000 Ghent, Belgium

† These authors contributed equally to this article.

* Corresponding author: Tom Van de Wiele, CMET – UGent. Coupure Links 653, B-9000 Ghent, Belgium. Phone: +32 9 264 59 76. Fax: + 32 9 264 62 48. E-mail: tom.vandewiele@ugent.be

Supplementary table 1

		A1		A2		A3		A4		A5	
		Lumen	Mucus	Lumen	Mucus	Lumen	Mucus	Lumen	Mucus	Lumen	Mucus
IV	Control	5.53 ± 0.00	-	4.93 ± 0.04	4.01 ± 0.47	7.04 ± 0.07	6.72 ± 0.03	6.40 ± 0.06	6.46 ± 0.00	7.43 ± 0.01	7.84 ± 0.03
	BP 25-3	5.73 ± 0.04	3.88 ± 0.00	5.33 ± 0.01	4.32 ± 0.21	7.09 ± 0.00	6.95 ± 0.04	7.09 ± 0.05	6.24 ± 0.40	7.82 ± 0.09	8.08 ± 0.05
	BP 1.20	5.98 ± 0.03	5.84 ± 0.04	4.97 ± 0.23	3.90 ± 0.00	6.50 ± 0.12	6.61 ± 0.03	6.82 ± 0.06	5.82 ± 0.38	7.44 ± 0.07	8.02 ± 0.01
	FP	5.99 ± 0.07	4.69 ± 0.30	5.67 ± 0.02	-	7.02 ± 0.00	6.24 ± 0.04	8.13 ± 0.04	6.55 ± 0.02	7.26 ± 0.03	7.54 ± 0.03
	MIX	5.65 ± 0.40	5.99 ± 0.00	3.91 ± 0.22	4.01 ± 0.30	6.90 ± 0.12	6.91 ± 0.01	7.41 ± 0.02	6.06 ± 0.06	7.82 ± 0.04	7.69 ± 0.06
XIVa	Control	4.02 ± 0.08	4.28 ± 0.10	3.96 ± 0.46	4.21 ± 0.02	9.03 ± 0.10	8.64 ± 0.08	8.24 ± 0.09	7.29 ± 0.00	8.46 ± 0.04	8.47 ± 0.07
	BP 25-3	3.51 ± 0.19	3.56 ± 0.08	4.05 ± 0.10	5.14 ± 0.07	9.14 ± 0.02	8.54 ± 0.05	8.46 ± 0.04	7.71 ± 0.04	8.55 ± 0.03	8.50 ± 0.10
	BP 1.20	4.45 ± 0.12	3.79 ± 0.53	3.41 ± 0.11	5.81 ± 0.06	8.74 ± 0.02	8.33 ± 0.05	8.41 ± 0.03	7.60 ± 0.04	8.47 ± 0.04	8.49 ± 0.07
	FP	4.30 ± 0.13	3.67 ± 0.12	4.80 ± 0.03	4.67 ± 0.27	9.26 ± 0.02	7.43 ± 0.09	8.32 ± 0.04	7.24 ± 0.07	8.40 ± 0.02	7.53 ± 0.08
	MIX	4.02 ± 0.04	5.07 ± 0.00	3.86 ± 0.05	4.98 ± 0.08	9.19 ± 0.01	8.60 ± 0.05	8.67 ± 0.03	7.35 ± 0.02	8.43 ± 0.02	7.93 ± 0.19
		R1		R2		R3		R4		R5	
		Lumen	Mucus	Lumen	Mucus	Lumen	Mucus	Lumen	Mucus	Lumen	Mucus
IV	Control	7.40 ± 0.09	6.60 ± 0.00	6.35 ± 0.14	6.23 ± 0.01	6.98 ± 0.09	7.11 ± 0.08	6.78 ± 0.02	7.04 ± 0.06	8.84 ± 0.03	8.63 ± 0.05
	BP 25-3	7.23 ± 0.01	6.08 ± 0.05	6.56 ± 0.02	6.70 ± 0.06	7.22 ± 0.26	7.25 ± 0.06	6.75 ± 0.00	6.81 ± 0.07	8.86 ± 0.03	8.39 ± 0.05
	BP 1.20	7.18 ± 0.04	6.37 ± 0.03	6.45 ± 0.06	6.53 ± 0.02	7.20 ± 0.03	6.57 ± 0.00	8.05 ± 0.02	7.20 ± 0.04	8.58 ± 0.05	7.59 ± 0.02
	FP	7.50 ± 0.07	6.91 ± 0.06	6.93 ± 0.28	6.82 ± 0.01	7.03 ± 0.06	7.19 ± 0.01	8.05 ± 0.02	7.48 ± 0.06	8.84 ± 0.05	8.84 ± 0.05
	MIX	7.21 ± 0.03	6.94 ± 0.06	6.47 ± 0.04	6.32 ± 0.04	7.30 ± 0.03	7.06 ± 0.04	7.97 ± 0.04	8.99 ± 0.04	8.67 ± 0.08	8.67 ± 0.08
XIVa	Control	9.05 ± 0.04	8.58 ± 0.01	9.17 ± 0.00	8.86 ± 0.04	8.52 ± 0.01	9.66 ± 0.07	9.38 ± 0.01	8.47 ± 0.05	8.99 ± 0.02	7.91 ± 0.05
	BP 25-3	9.12 ± 0.05	8.16 ± 0.02	9.12 ± 0.03	9.59 ± 0.05	8.64 ± 0.04	9.28 ± 0.14	9.30 ± 0.02	8.28 ± 0.02	8.88 ± 0.06	8.37 ± 0.05
	BP 1.20	9.01 ± 0.04	8.71 ± 0.03	9.06 ± 0.01	10.54 ± 0.16	8.51 ± 0.07	8.09 ± 0.30	9.17 ± 0.04	8.36 ± 0.02	8.54 ± 0.04	-
	FP	9.06 ± 0.06	8.46 ± 0.00	9.33 ± 0.01	10.02 ± 0.09	8.55 ± 0.01	8.51 ± 0.05	9.17 ± 0.04	8.09 ± 0.02	8.67 ± 0.02	8.67 ± 0.02
	MIX	9.08 ± 0.04	9.02 ± 0.06	9.28 ± 0.02	9.21 ± 0.04	8.63 ± 0.04	7.86 ± 0.03	8.81 ± 0.01	-	8.42 ± 0.01	8.42 ± 0.01

Supplementary Table 1 *Concentration of Clostridial cluster IV and cluster XIVa in log copies/mL in lumen- and mucus-associated microbiota of five active CD (A) and five remissive CD (R) samples 65h after start incubation and treatment. Values are averages of technical replicates (n=3) ± standard deviation. Light grey boxes indicate a higher concentration in treated sample compared to non-treated control; dark grey boxes indicate a difference with control of at least 1 log unit.*

Supplementary Table 2

Label	Gender	Age	Location of the disease	WBC feces	CRP blood (mg/L)	Medical therapy
**A 1**	F	36	Colonic	21.164	-	Immunosuppressive drug
**A 2**	F	57	Ileocolonic	-	-	Mesalamine
**A 3**	F	28	Ileocolonic	-	15,9	Immunosuppressive drug
**A 4**	F	13	Ileocolonic	115.506	30,8	Nutritional therapy, Immunosuppressive
**A 5**	M	14	Pancolitic	511.341	26,9	none
**R 1**	F	-	-	-	-	Anti-tumor necrosis factor alpha
**R 2**	M	20	-	4.503	-	Immunosuppressive drug; Mesalamine
**R 3**	F	26	Ileal	3.364	-	none
**R 4**	F	43	-	5.720	-	none
**R 5**	F	64	-	3.598	-	Immunosuppressive drug

Supplementary Table 2 *Metadata of Crohn's disease patients used for fecal sample collection. WBC: White Blood Cell count in fecal suspension. CRP: C-Reactive Protein levels blood.*

Supplementary table 3

Target group	Short name	Primer	Use	Sequence (5'→3')	Annealing T (°C)	Fragment size	Reference
Clostridium leptum group	Clept group/ Cluster IV	Sg-Clept-F	PCR-DGGE	CGCCCGCCGCGCGCGGCGG GCGGGGCGGGGGCACGGG GGG **GCACAAGCAGTGGAGT**	50	239 bp	[50]
		Sg-clept-R3-GC		CTT CCT CCG TTT TGT CAA			
		Clept-f	qPCR	GCA CAA GCA GTG GAG T (Tm 49)	50	239 bp	[51]
		Clept-R3		CTTCCTCCGTTTTGTCAA (Tm 53)			
Eubacterium rectale-Blautia coccoides group	Erec group/ Cluster XIVa	Cocc-f	PCR-DGGE	AAA TGA CGG TAC CTG ACT AA	54	440 bp	[52]
		Cocc-r+GC		CGCCCGGGGCGCGCCCCGG GCGGGGCGGGGGCACGGG GGG **CTT TGA GTT TCA TTC TTG CGA A**			[53]
		g-Ccoc-f	qPCR	AAA TGA CGG TAC CTG ACT AA (Tm 51)	50	440 bp	[51]
		g-Ccoc-r		CTT TGA GTT TCA TTC TTG CGA A (Tm 58)			

Supplementary Table 3 *Primers used for PCR-DGGE and qPCR*

Supplementary table 4

Protocol	Polyacrylamide % (w/v)	Gradient[1]	DGGE system	Run conditions
Total bacteria	8%	45% - 60%	PhorU Ingeny	120 V – 60°C – 16h
Clostridial cluster IV	8%	30% - 52,5 %	DCode Bio-Rad	200 V – 60°C – 4h
Clostridial cluster XIVa	6%	38% - 60%	PhorU Ingeny	120 V – 60°C – 16h

[1] Denaturating gradient: 100% is 7 M urea and 40% formamide

Supplementary Table 4 *DGGE conditions.*

Supplementary table 5

Patient	Sample	pH	Total SCFA (mM)	Acetate (mM)	Propionate (mM)	Butyrate (mM)
**A 3**	CON	6.45	49.4	29.4	9.2	8.5
	BP 25-3	6.27	40.4	23.2	7.9	7.9
	MIX	6.31	40.5	23.2	6.9	10.0
**A 4**	CON	6.23	14.3	11.0	3.2	0.0
	BP 25-3	6.31	18.0	12.5	5.1	0.3
	MIX	6.29	18.2	11.1	3.7	3.2
**A 5**	CON	6.17	38.2	23.0	8.3	5.4
	BP 25-3	6.19	42.3	26.4	8.7	5.6
	MIX	6.28	32.2	16.9	7.2	7.1
**R 2**	CON	6.20	41.1	29.6	6.5	4.7
	BP 25-3	6.08	42.4	30.9	7.0	4.3
	MIX	6.16	40.5	26.6	7.0	6.6
**R3**	CON	-	38.2	23.1	7.3	5.7
	BP 25-3	6.37	41.2	24.9	8.1	6.0
	MIX	6.33	41.4	23.8	8.0	8.0
**R4**	CON	6.13	38.0	22.8	8.7	6.3
	BP 25-3	6.26	35.0	21.6	8.2	5.1
	MIX	6.27	41.1	23.5	9.2	8.3

Supplementary Table 5 *Characteristics of undiluted supernatant samples of fed batch system of CD microbiota incubated for 42h and used in Caco-2 cell assays.(- : sample not analyzed)*
